# Alterated gene expression in dilated cardiomyopathy after left ventricular assist device support by bioinformatics analysis

**DOI:** 10.3389/fcvm.2023.1013057

**Published:** 2023-03-17

**Authors:** Ying Wei, Hao Cao, Yuan-Yi Peng, Bo Zhang

**Affiliations:** ^1^ Department of Ultrasound in Medicine, Shanghai East Hospital, School of Medicine, Tongji University, Shanghai, China; ^2^ Department of Cardiovascular Surgery, Shanghai East Hospital, School of Medicine, Tongji University, Shanghai, China; ^3^ Engineering Research Center of Artificial Heart and Heart Failure Medicine, Shanghai, China

**Keywords:** dilated cardiomyopathy, left ventricular assist device (LVAD), hub gene, biomarker, bioinformactics analysis

## Abstract

**Introduction:**

Heart transplantation is the best treatment for end-stage dilated cardiomyopathy (DCM). Left ventricular assist device (LVAD) support is becoming more prevalent and may delay heart transplantation. Gene expression of the left ventricular myocardium usually changes following LVAD implantation. In this study, we aimed to identify potential biomarkers to determine the prognosis of patients with DCM after receiving LVAD support.

**Methods:**

We extracted microarray datasets from Gene Expression Omnibus (GEO), including GSE430 and GSE21610. There were 28 paired DCM samples in the GSE430 and GSE21610 profiles. Differentially expressed genes (DEGs) were identified at LVAD implantation and heart transplantation. DEGs were annotated according to Gene Ontology (GO) and analyzed according to the Kyoto Encyclopedia of Genes and Genomes (KEGG) pathway enrichment analysis. A protein–protein interaction (PPI) network was constructed. The top 10 crucial genes were predicted using Cytoscape plugin CytoHubba in conformity with the network degree algorithm. The levels of gene expression and the diagnostic values of crucial genes were confirmed in the clinical datasets.

**Results:**

The 28 DEGs were clustered into the GSE datasets. GO annotations and KEGG pathway enrichment analyses revealed that inflammation might be involved. They were associated with correlative inflammation. Combined with PPI networks, these results revealed CytoHubba's top 10 hub genes, including *CCL2*, *CXCL12*, *CXCL1*, *CTGF*/*CCN2*, *CX3CR1*, *POSTN*, *FKBP5*, *SELE*, *AIF1*, and *BMP2*. Among them, *CCL2*, *CXCL12*, *FKBP5*, and *BMP2* might be considered prognostic and diagnostic biomarkers after LVAD support and have confirmed their validity in clinical datasets. The area under the curve of the four main hub genes was more than 0.85, indicating high diagnostic ability and good prognosis for patients with DCM with LVAD implantation. However, a significant effect of *CCL2*, *CXCL12*, *FKBP5*, and *BMP2* expression was not observed on the left ventricular end-diastolic diameter (LVEDD), left ventricular ejection fraction (LVEF), cardiac index (CI), or support time of LVAD.

**Conclusion:**

*CCL2*, *CXCL12*, *FKBP5*, and *BMP2* could be potential gene biomarkers for patients with DCM after LVAD support. These findings provide critical clues for the therapeutic management of patients with DCM and LVADs. LVEDD, LVEF, CI, and support time of LVAD were not correlated with the expression of these hub genes.

## 1. Introduction

Dilated cardiomyopathy (DCM) is a common manifestation of end-stage heart disease characterized by left ventricle dilation and impaired contractility without secondary causes (1). High mortality of DCM suggests that heart transplantation is the best treatment for end-stage DCM. However, a shortage of heart donors increases waiting times for patients to undergo heart transplantation. Left ventricular assist device (LVAD) is becoming a bridge to heart transplantation or a destination therapy for patients with DCM and end-stage congestive heart failure (CHF) (2, 3). The use of LVAD significantly decreases mortality and improves the quality of life of patients with CHF (4, 5). Some patients’ symptoms of heart failure improve after implantation of a ventricular assist device, which can delay the time of heart transplantation or even prevent it.

However, due to the different support times of LVAD among individuals, we hypothesized that specific biomarkers could predict the prognosis after implantation of assist devices and intervene early. Gene expression in the left ventricular myocardium changed after LVAD implantation (6), and changes in genes may be another manifestation of CHF (7). Changes in gene expression could be considered as a method to detect the progression of the disease and cardiac remodeling after LVAD implantation. Some studies have investigated molecular levels after mechanical unloading. However, there has been little agreement on the molecular mechanisms of LVAD support. A better understanding of the gene expression patterns would provide a new perspective on the potential prognosis and treatment of DCM. Our study indicates that *CCL2*, *CXCL12*, *FKBP5*, and *BMP2* might be related to clinical outcomes. These findings provide critical clues to the therapeutic strategies for DCM with end-stage CHF.

## 2. Materials and methods

### 2.1. Data source

Gene expression profiles [GSE430 (6) and GSE21610 (7)] were obtained from the National Center for Biotechnology Information (NCBI) Gene Expression Omnibus (GEO) database (http://www.ncbi.nlm.nih.gov/geo/). The study participants were patients with DCM who did not experience secondary myocardial diseases, such as ischemia, valvulopathies, and congenital disorders. The GSE430 dataset consists of seven paired DCM and end-stage heart failure samples with LVAD support. The GSE21610 profile contains 21 paired samples of DCM and 9 paired samples from ischemic cardiomyopathy (ICM) with LVAD support. We analyzed only 28 paired DCM samples from the GSE430 and GSE21610 datasets. All data are freely available from GEO. The GSE430 dataset was based on the GPL96 platform [(HG-U133A) Affymetrix Human Genome U133A Array], while the GSE21610 dataset was based on the GPL570 platform [(HG-U133_Plus_2) Affymetrix Human Genome U133 Plus 2.0 Array]. This study did not have a relation with any experiments conducted by any authors on humans or animals. No ethical approval was required, given the lack of human or animal subjects in this study.

### 2.2. Identification of differentially expressed genes

GEO2R, an online tool (http://www.ncbi.nlm.nih.gov/geo/geo2r/) with an R-based web (R 3.2.3, Biobase 2.30.0, GEOquery 2.40.0, limma 3.26.8), was used to analyze GEO data to screen out DEGs between pre-LVAD and post-LVAD groups in the dataset. A false discovery rate *P* value <.05 and |log2fold change (FC) |≥1 were set as the standard for identifying DEGs. Log2FC ≥1 for upregulated genes and log2FC ≤ −1 for downregulated genes were considered statistically significant. Overlapping DEGs were analyzed by a Venn diagram. Venn diagrams and heatmaps of DEGs were produced using TBtools, an integrative toolkit for interactive biological data (8). A free online data analysis tool, Sangerbox, was used to analyze volcano data (http://www.sangerbox.com/tool) (9), and the gene names of the top 10 upreregulated DEGs and downreregulated DEGs were indicated in the volcano plots.

### 2.3. GO and KEGG pathway analysis

GO annotation and KEGG pathway analyses were conducted to determine the functions of DEGs on David (http://david.ncifcrf.gov) (10, 11), an online tool for gene annotation and analysis. Biochemical processes (BPs), cellular components (CCs), and molecular functions (MFs) were included in the GO annotation analysis. *P* value < .05 was used as an enrichment threshold for GO terms and KEGG pathway segments. Sangerbox was used to visualize GO enrichment and KEGG pathway analyses (9).

### 2.4. PPI network construction and hub gene screening

Protein—protein interaction (PPI) networks help construct a functional protein system by combining all the protein-coding genes into one huge biological network. In the study, by mapping DEGs into the database, we found important protein pairs with a minimum required interaction score of 0.4 and removed the hidden disconnected nodes. An evaluation of the PPI network of DEGs was conducted using the Search Tool for the Retrieval of Interacting Genes (STRING; http://string-db.org/), which integrates associations for the PPI network (12–14). Using Cytoscape software version 3.9.1, the PPI network was visualized by calculating the degree of protein connectivity. CytoHubba is a plugin for Cytoscape that uses multiple topological algorithms to rank nodes in a PPI network. We used Cytohubba software to identify the central genes.The top 10 hub genes were selected using the degree method because they perform better in predicting essential proteins accurately. The hub genes were analyzed using the GeneMANIA online database (https://genemania.org/).

### 2.5. Impact on the characterization of clinical data

The expression of the top 10 hub genes in the pre- and post-LVAD groups was analyzed using GraphPad Prism 8 software. We also studied the relationship between the expression of the top 10 hub genes and clinical information.

### 2.6. Statistical analysis

GraphPad Prism 8 software was used to perform the statistical analysis. Measures of continuous variation were expressed as means ± standard deviations (SDs), and categorical data were expressed as numbers or percentages. Continuous variables were compared by Student's *t*-test if the data were normally distributed; otherwise, they were compared by nonparametric statistics. Categorical data were analyzed by the chi-square test. Statistical significance was set at *P* < .05, and all statistical analyses were two-sided.

## 3. Results

### 3.1. Screening for DGES

**Figure 1:**
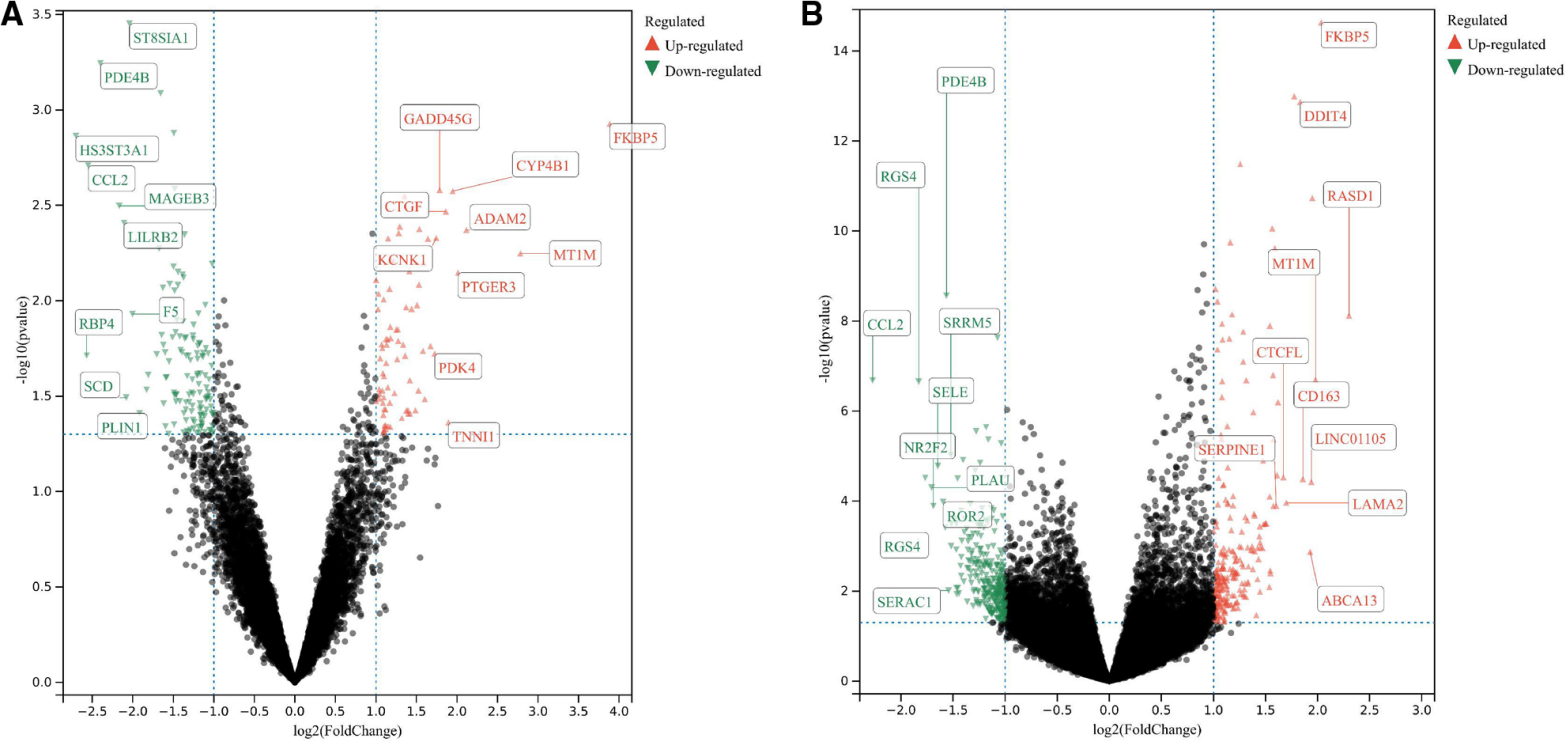
(**A**) Volcanic map of the GSE430 dataset; (**B**) volcanic map of the GSE21610 dataset. All genes were presented in volcano plots. Red ones were upregulated DEGs, green ones were downregulated DEGs, and black ones were the rest of the genes. The name of the top 10 upregulated and downregulated DEGs are shown in the volcanic plots. DEGs: differentially expressed genes.

**Figure 2:**
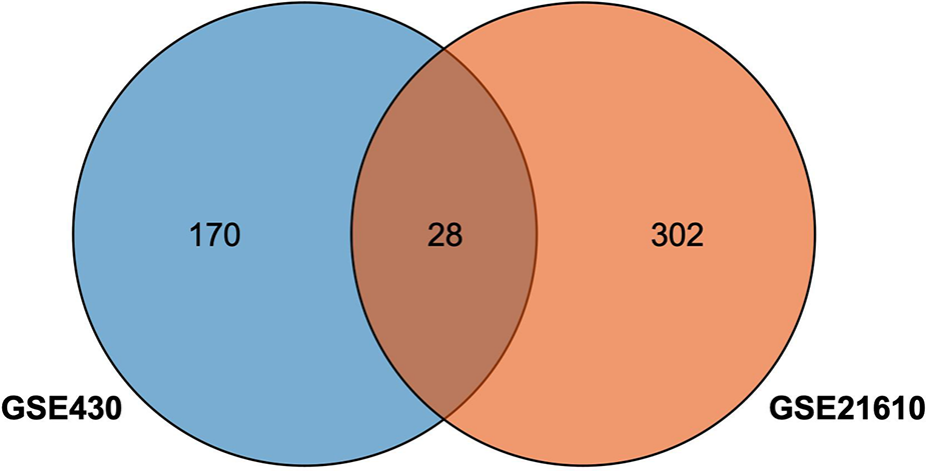
Venn diagram of overlapping of the differentially expressed genes in the datasets.

**Figure 3:**
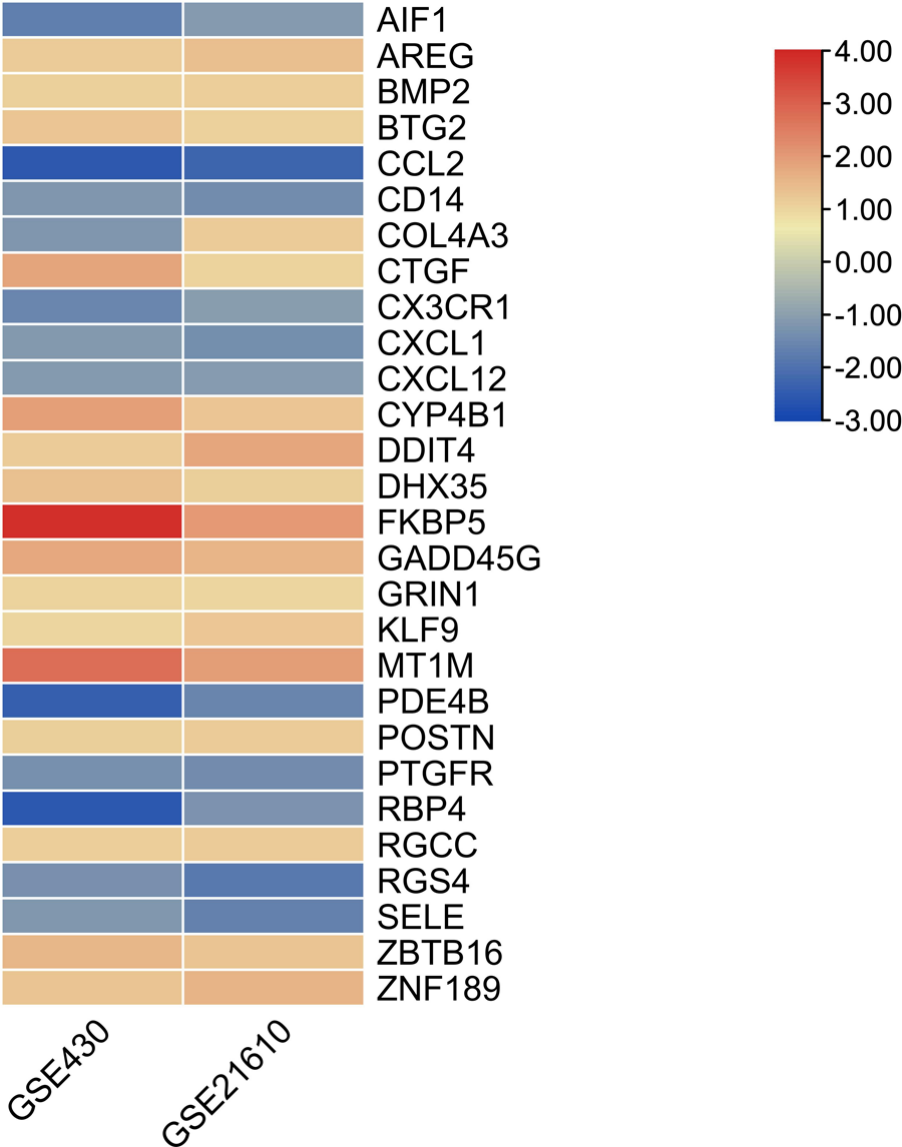
Heatmap of the differentially expressed genes in the datasets.

The GSE430 and GSE21610 datasets were selected and screened for DEGs using GEO2R. By screening using the standard of significance (*P* value <.05 and |log2FC| ≥ 1), GSE430 identified 198 DEGs (76 upregulated and 122 downregulated) and GSE21610 identified 330 DEGs (151 upregulated and 179 downregulated). All genes are presented in volcano plots (Figures 1A,B). Volcano analysis was performed using Sangerbox tools, an online data analysis platform that is free. The names of the top 10 upregulated and downregulated DEGs are shown in the volcano plots. There were 28 DEGs in the overlapping dataset (Figure 2). A heatmap of DEGs in the datasets is shown (Figure 3).

### 3.2. GO and KEGG functional enrichment analyses

**Figure 4:**
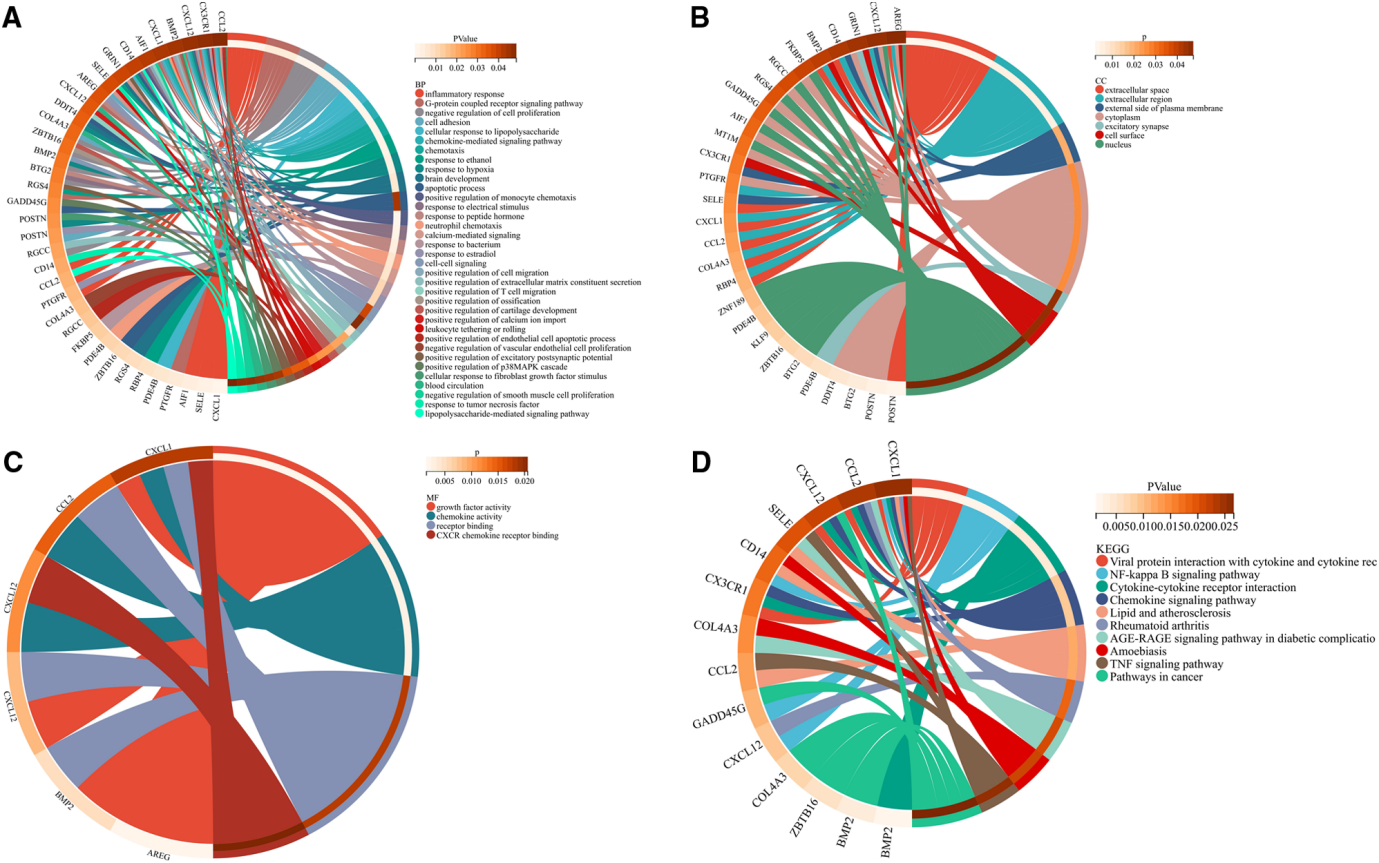
Common differentially expressed genes according to the Gene Ontology and Kyoto Encyclopedia of Genes and Genomes databases. (**A**) Biological process of differentially expressed genes. (**B**) Cellular component of differentially expressed genes. (**C**) Molecular function of differentially expressed genes. (**D**) Kyoto Encyclopedia of Genes and Genomes pathway of differentially expressed genes.

Functional enrichment analyses of the DEGs were performed using the Database for Annotation, Visualization, and Integrated Discovery (DAVID; http://www.david.org). In the GO analysis results, BP, MF, and CC appeared to be significantly enriched in DEGs. BP (Figure 4A) analysis majored in the inflammatory response, G-protein-coupled receptor signaling pathway, negative regulation of cell proliferation, cell adhesion, cellular response to lipopolysaccharide, etc. In CC (Figure 4B), these DEGs were associated with extracellular space, extracellular region, external side of the plasma membrane, cytoplasm, excitatory synapse, cell surface, and nucleus. In MF (Figure 4C), the analysis indicated growth factor activity, chemokine activity, receptor binding, and CXCR chemokine receptor binding. KEGG pathway analysis showed that the DEGs were significantly enriched in viral protein interaction with cytokine and cytokine receptor, NF-kappa B signaling pathway, cytokine–cytokine receptor interaction, chemokine signaling pathway, lipid and atherosclerosis, rheumatoid arthritis, AGE-RAGE signaling pathway in diabetic complications, amoebiasis, TNF signaling pathway, and pathways in cancer (Figure 4D). The GO enrichment and KEGG analyses were visualized using Sangerbox tools.

### 3.3. Building PPI networks and identifying hub genes

**Figure 5:**
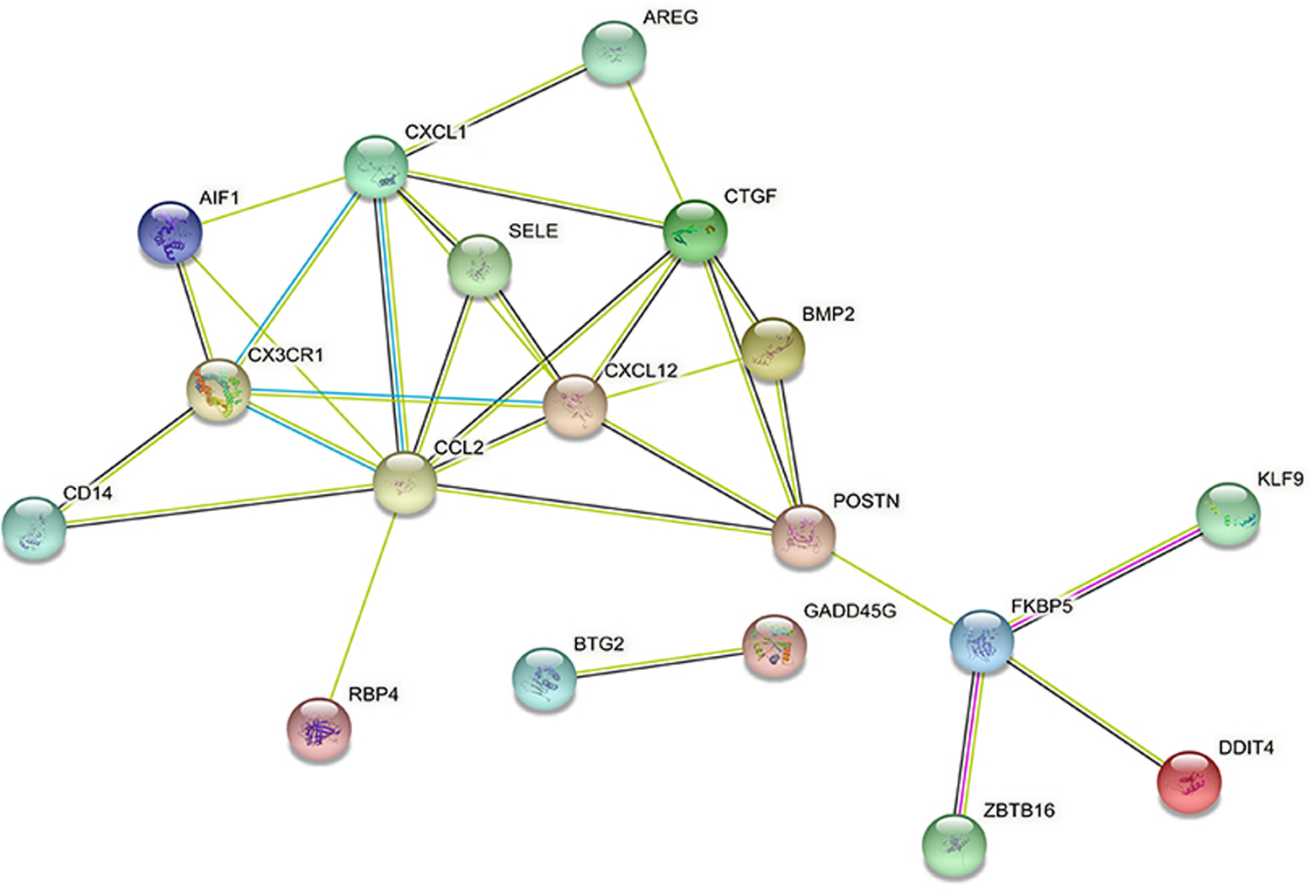
Protein–protein interaction network of differentially expressed genes created by STRING.

**Figure 6:**
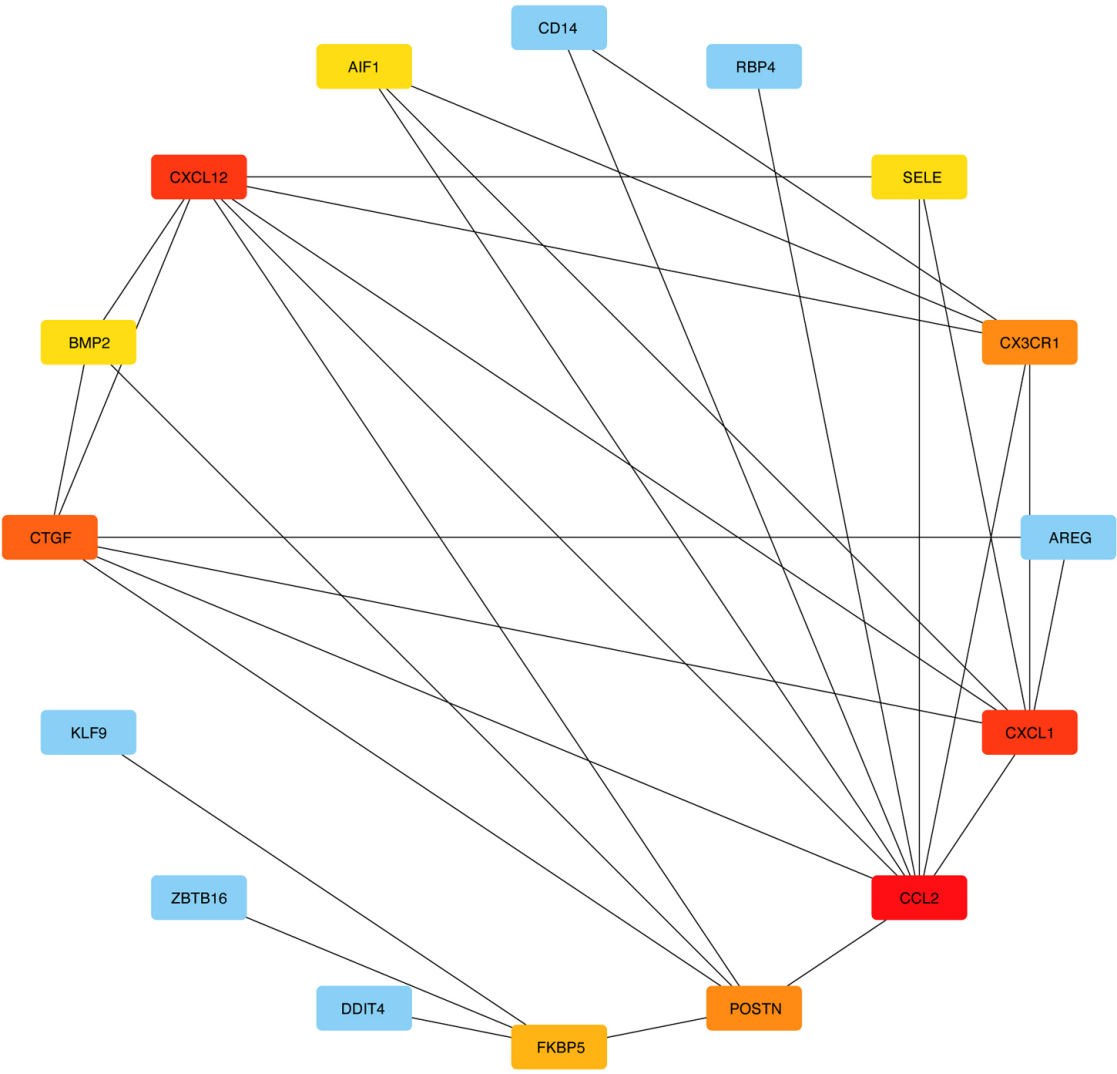
Network of differentially expressed genes created by Cytoscape.

**Figure 7:**
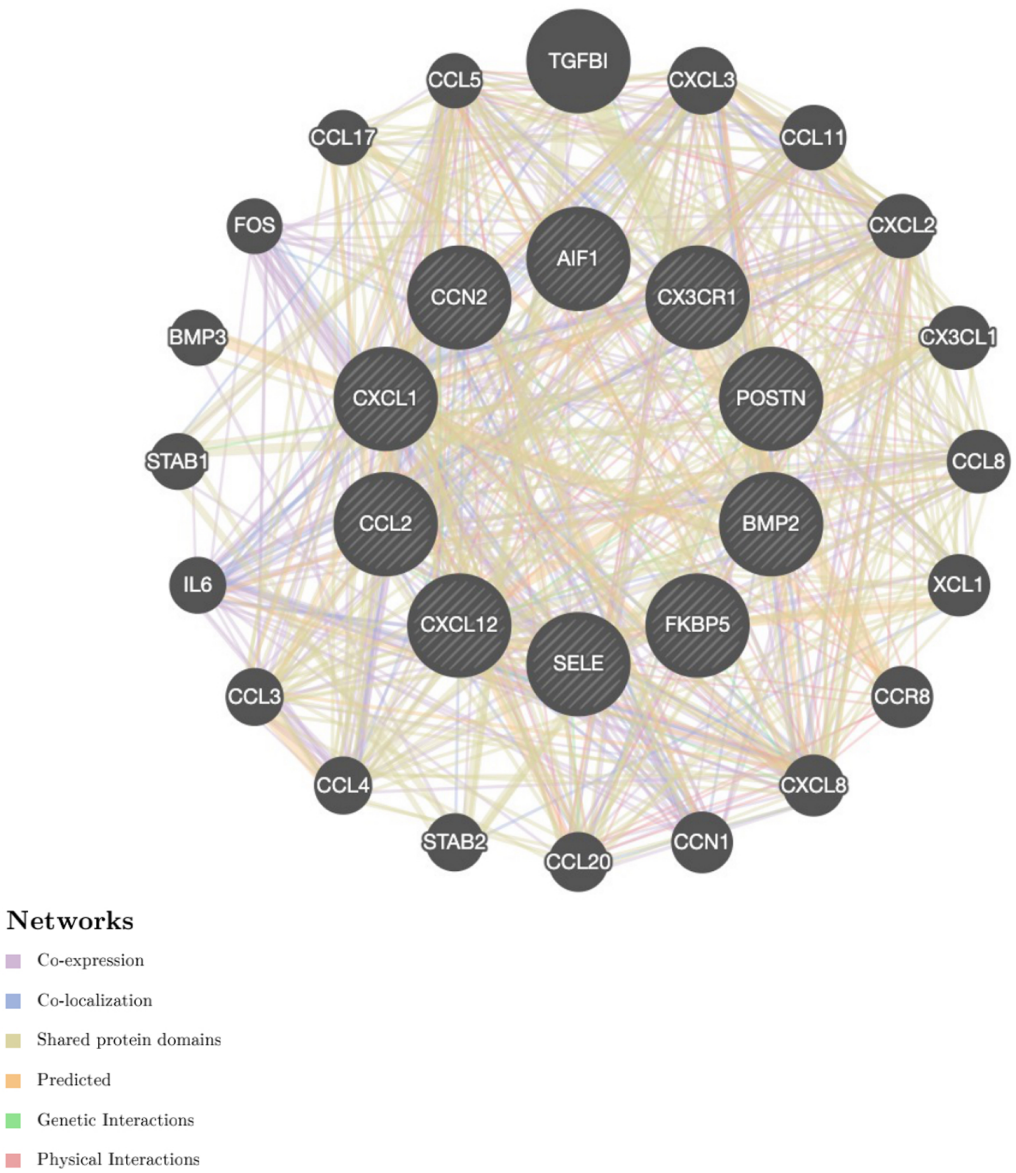
Protein–protein interaction network of the top 10 hub genes. Inner circles represent the hub genes, and outer circles correspond to GeneMANIA-proposed hub genes.

DEGs were evaluated based on the connectivity degree of the PPI network, which was built using the STRING database (Figure 5). The top 10 genes were considered hub genes according to the degree algorithms in the Cytoscape soft. Among the significant genes, those with the highest connectivity were identified, and the relationships between the hub genes are shown in Figure 6. The top 10 hub genes were C–C motif chemokine ligand 2 (CCL2), C–X–C motif chemokine ligand 12 (CXCL12), C–X–C motif chemokine ligand 1 (CXCL1), connective tissue growth factor (CTGF/CCN2), C–X3–C motif chemokine receptor 1 (CX3CR1), periostin (POSTN), FK506-binding protein 5 (FKBP5), selectin E (SELE), allograft inflammatory factor 1 (AIF1), and bone morphogenetic protein 2 (BMP2). The results of the top 10 hub genes’ eight algorithms are presented in Table 1. Table 2 presents a description of the top 10 hub genes. The PPI network of the top 10 hub genes was constructed using GeneMania (http://genemania.org/) (Figure 7). This indicated a relationship between the top 10 hub genes and other related genes. *CTGF* is also known as cellular communication network factor 2 (CCN2). *CNN2* represents the *CTGF*, as shown in Figure 7. Among them, *CTGF*, *POSTN*, *FKBP5*, *SELE*, *AIF1*, and *BMP2* were upregulated, while *CCL2*, *CXCL12*, *CXCL1*, and *CX3CR1* were downregulated.

**Table 1:** Results of the top 10 hub genes’ eight algorithms.

Hub gene	MCC	Degree	Bottleneck	Closeness	Eccentricity	EPC	MNC	Radiality
*CCL2*	33	9	4	11.5	0.2963	6.898	8	3.31852
*CXCL12*	30	7	4	10.5	0.2963	6.672	7	3.2
*CXCL1*	26	7	3	10.08333	0.22222	6.662	7	2.96296
*CTGF*	20	6	3	10	0.2963	6.418	6	3.14074
*CX3CR1*	14	5	2	9.08333	0.22222	5.964	5	2.84444
*POSTN*	13	5	5	10	0.44444	6.027	4	3.25926
*FKBP5*	4	4	4	8.33333	0.2963	3.449	1	2.78519
*SELE*	6	3	1	8.08333	0.22222	5.129	3	2.72593
*AIF1*	6	3	1	8	0.2963	5.037	3	2.78519
*BMP2*	6	3	1	7.91667	0.22222	5.095	3	2.66667

**Table 2:** Description of the top 10 hub genes.

Gene symbol	Rank degree	Gene ID	Gene description	Expression
CCL2	1	6,347	C–C motif chemokine ligand 2	Downregulated
CXCL1	2	2,919	C–X–C motif chemokine ligand 1	Downregulated
CXCL12	2	6,387	C–X–C motif chemokine ligand 12	Downregulated
CTGF	4	1,490	Connective tissue growth factor	Upregulated
CX3CR1	5	1,524	C–X3–C motif chemokine receptor 1	Downregulated
POSTN	5	10,631	Periostin	Upregulated
FKBP5	7	2,289	FK506 binding protein 5	Upregulated
SELE	8	6,401	Selectin E	Upregulated
AIF1	8	199	Allograft inflammatory factor 1	Downregulated
BMP2	8	5,166	Bone morphogenetic protein 2	Upregulated

### 3.4. Impact of hub genes on the characterization of clinical data

**Figure 8:**
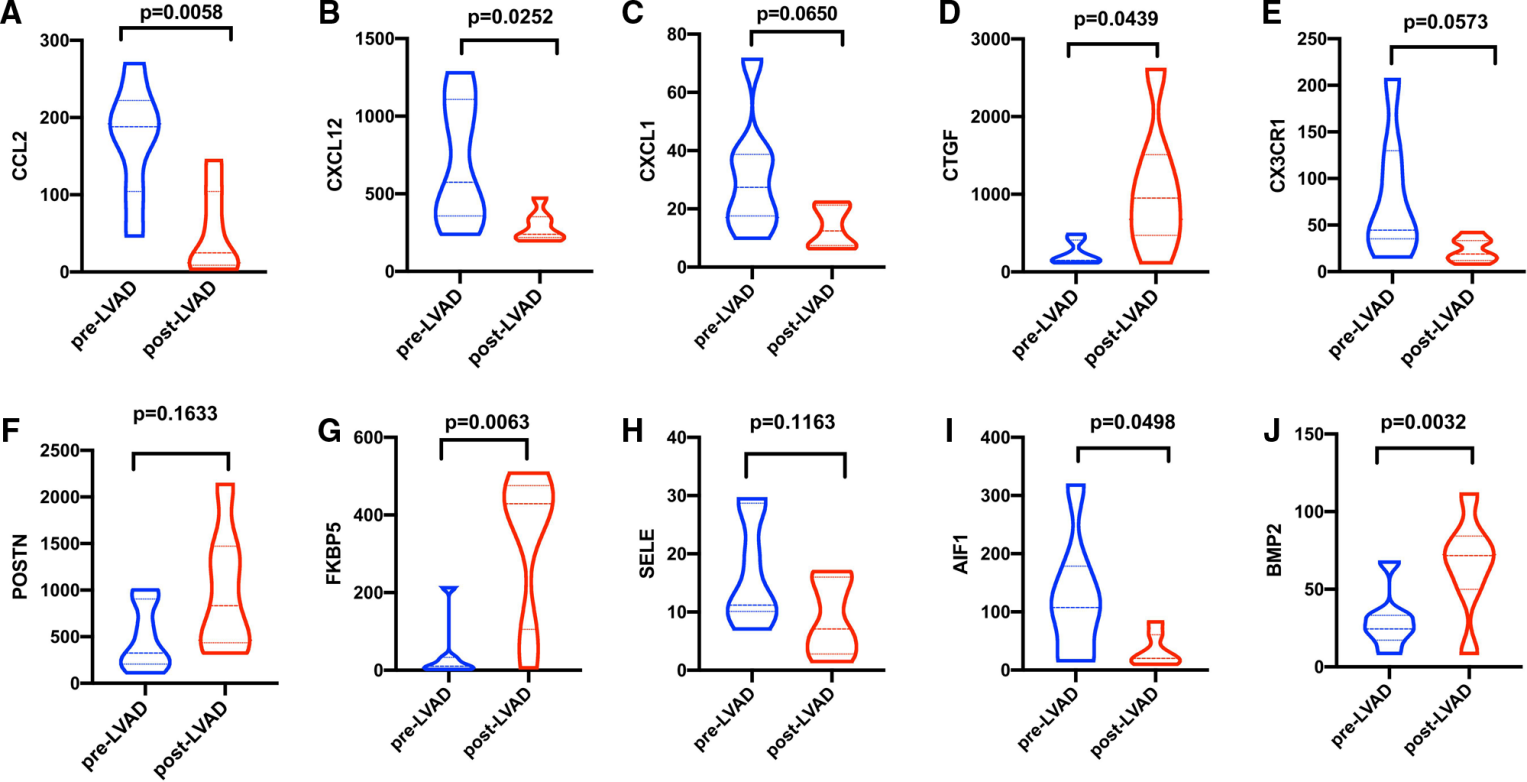
(**A–J**) Expressions of top 10 hub genes between pre-LVAD and post-LVAD groups in GSE430. The blue ones were pre-LVAD groups. The red ones were post-LVAD groups.

**Figure 9:**
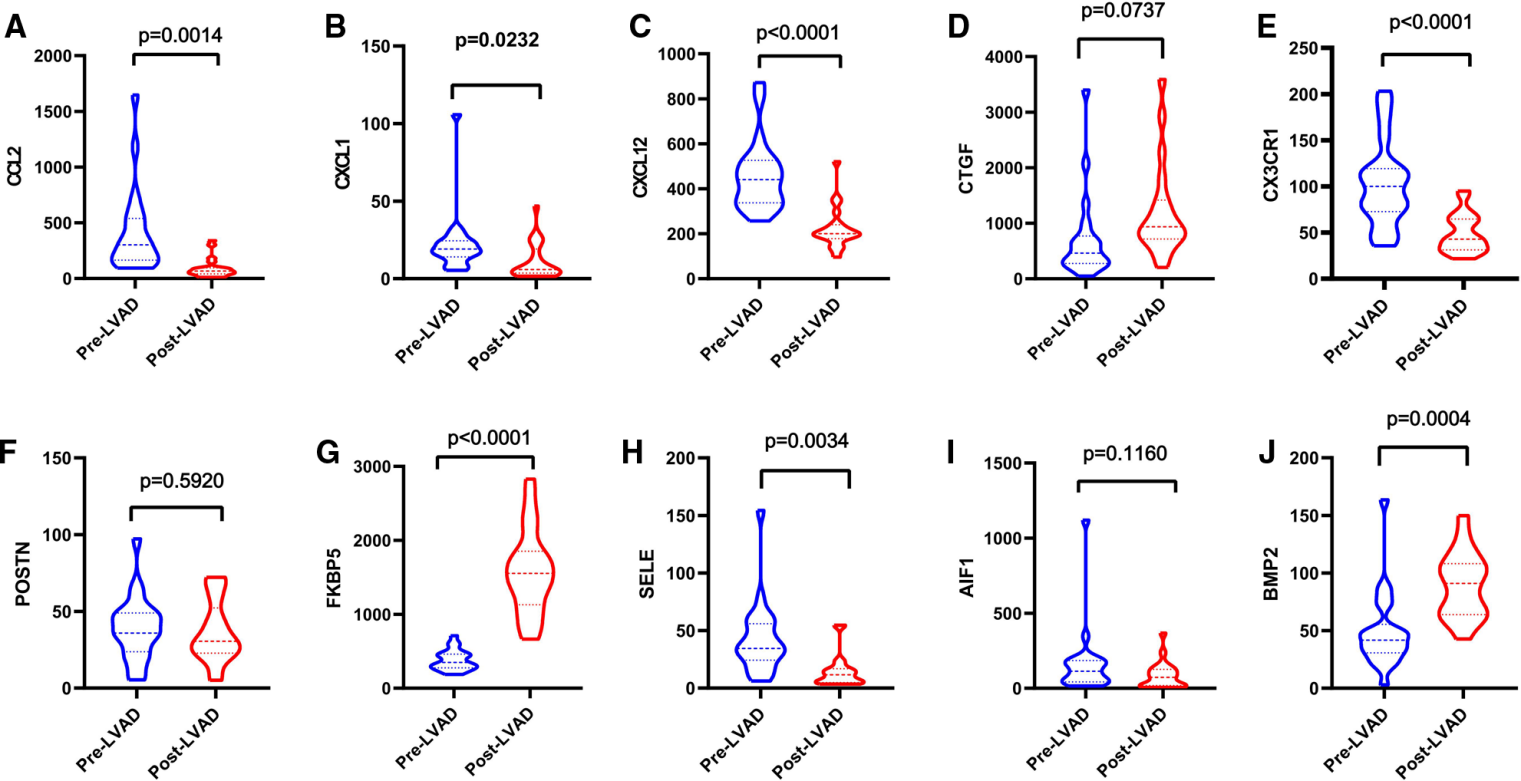
(**A–J**) Expressions of top 10 hub genes between pre-LVAD and post-LVAD groups in GSE21610. The blue ones were pre-LVAD groups. The red ones were post-LVAD groups.

**Figure 10:**
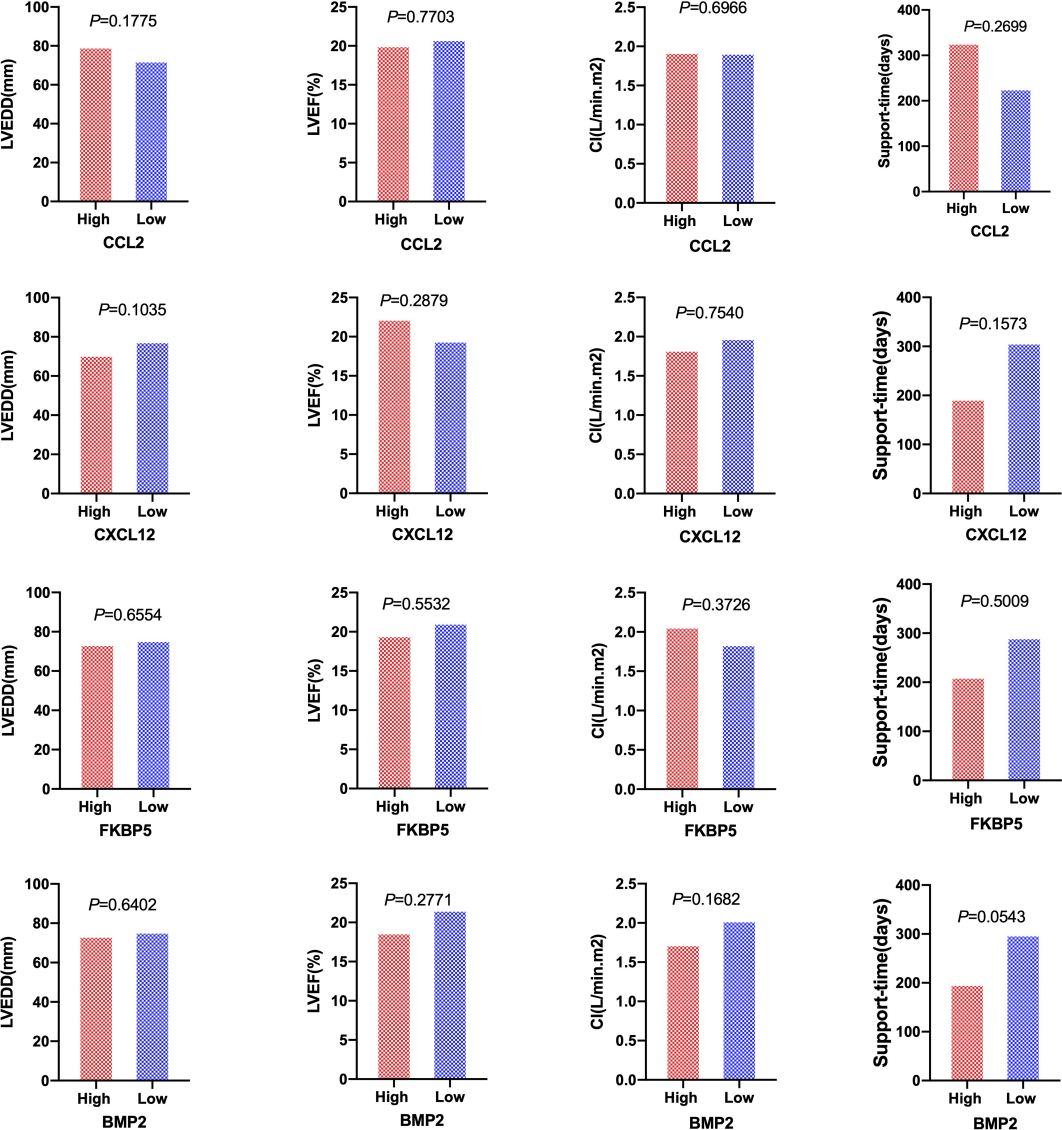
Patients of LVEDD, LVEF, CI, and the support time of LVAD between high-expression groups and low-expression groups of *CCL2*, *CXCL12, FKBP5*, and *BMP2* hub genes. High and rRed ones represented high-expression groups. The low and blue ones represented low-expression groups. LVEDD, left ventricular end-diastolic diameter; LVEF, left ventricular ejection fraction; CI, cardiac index.

**Figure 11:**
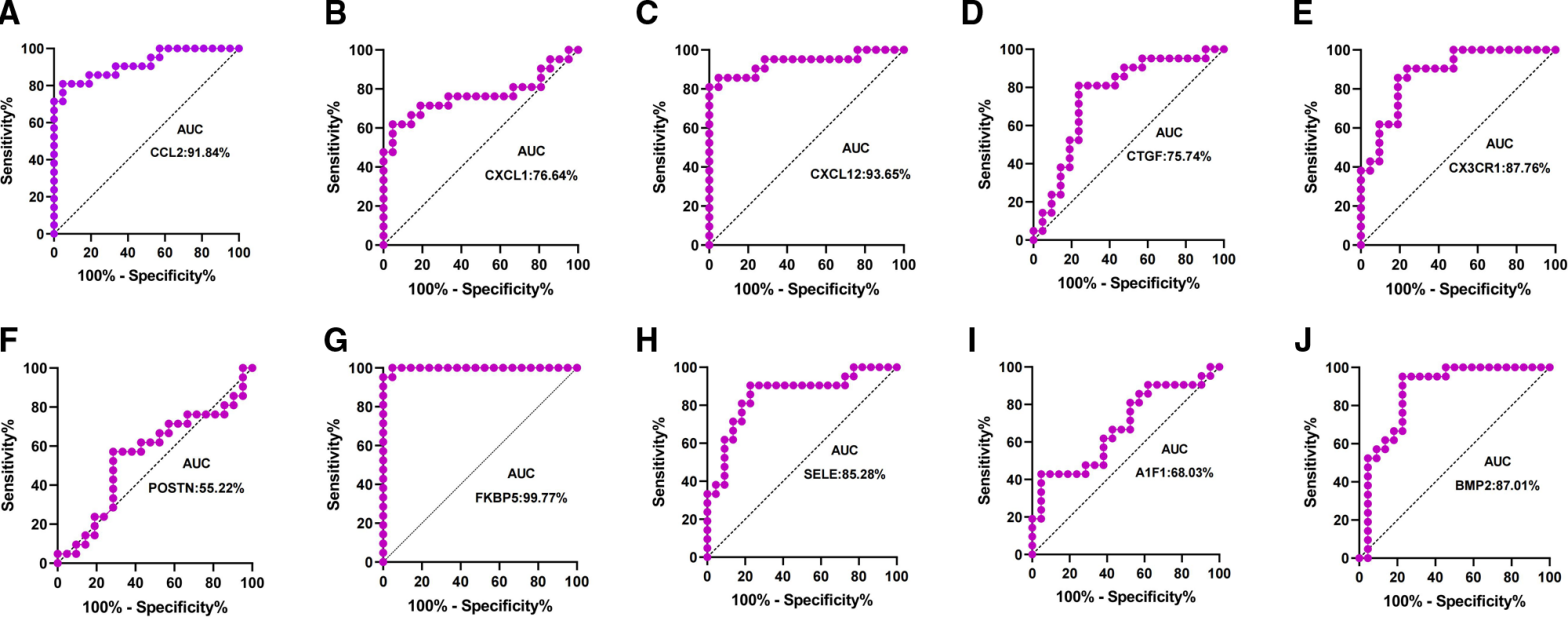
(**A–J**) ROC curves for top 10 hub genes, respectively.

Patients with DCM obtained from the GSE430 and GSE21610 datasets received LVAD support and heart transplantion. The characteristics of clinical data are presented in Table 3. The expression of the top 10 hub genes was assessed between pre- and post-LVAD groups in both datasets (Figures 8, 9). Only *CCL2*, *CXCL12*, *FKBP5*, and *BMP2* hub genes were significant in both clinical data (*P *< .05). A comparison of clinical data between the high and low expression levels of *CCL2*, *CXCL12*, *FKBP5*, and *BMP2* hub genes was conducted. There was no statistical difference between the different expression levels of the four hub genes and left ventricular end-diastolic diameter (LVEDD), left ventricular ejection fraction (LVEF), cardiac index (CI), or LVAD support time (*P *> 0.05) (Figure 10). However, LVEF and CI were significantly different between the GSE430 and GSE21610 datasets (*P* < 0.05) (Table 3). *CCL2*, *CXCL12*, *CX3CR1*, *FKBP5*, *SELE*, and *BMP2* demonstrated powerful diagnostic ability, with an area under the curve (AUC) > 0.85. AUCs for *CCL2*, *CXCL12*, and *FKBP5* were >0.90 (Figure 11).

**Table 3:** Characteristics of clinical data of patients with DCM.

	All patients (*n* = 28)	GSE430 (*n* = 7)	GSE21610 (*n* = 21)	*P* value
Age (years)	48.729 ± 12.01	47.43 ± 7.96	48.57 ± 13.08	0.8351
Male *n* (%)	24 (85.71)	5 (71.43)	19 (90.48)	0.2530
LVEDD (mm)	75.48 ± 11.38	75.14 ± 10.96	75.61 ± 11.54	0.9306
LVEF (%)	20.58 ± 6.77	15.71 ± 4.16	22.37 ± 6.67	0.0205*
CI	1.92 ± 0.56	1.46 ± 0.14	2.09 ± 0.95	0.0089*
Support time (days)	264.71 ± 198.84	193.86 ± 141.33	288.33 ± 209.35	0.5930

DCM, dilated cardiomyopathy; LVEDD, left ventricular end-diastolic diameter; LVEF, left ventricular ejection fraction; CI, cardiac index.

* *P *< 0.05: significant difference between GSE430 and GSE21610.

## 4. Discussion

Heart transplantation is the best treatment for advanced heart failure caused by DCM. LVAD is increasingly being used as a bridge to transplantation, recovery, or destination therapy (2, 3, 15) and is a sustainable alternative to heart transplantation. Implantation of LVAD can slow down the progress of heart failure and improve the symptoms of heart failure. In addition, it would decrease mortality and improve the quality of life of patients (4, 5). It has been proven that LVAD can lead to the reduction of ventricular diameter, reduction of LV mass, and improvement of cardiac ejection fraction (16, 17), and patients may experience different pathophysiological processes after LVAD support. Mechanical unloading induced significant changes in myocardial gene expression (18). Due to the different support times of LVAD in different individuals, we expect to find some biomarkers to predict the prognosis of heart failure after LVAD support and intervention as soon as possible. Some evidence indicates that the changes in gene expression in the left ventricular myocardium may be another manifestation of CHF following LVAD implantation (6, 7).

In this study, gene expression profiles of pre- and post-LVAD from DCM populations were compared using comprehensive bioinformatics. All the patients underwent cardiac transplantation after LVAD support. We screened 16 upregulated and 12 downregulated DEGs using GO and KEGG analyses. GO analysis of DEGs revealed the specific mechanisms of myocardial function after LVAD support and indirectly suggested that some molecular mechanisms might be involved in the LVAD support. However, further studies are required to confirm and validate these findings. Gene ontology analysis suggested that several mechanisms of inflammatory response activation including inflammatory response, G-protein coupled receptor signaling pathway, negative regulation of cell proliferation, cell adhesion, and cellular response to lipopolysaccharides in BP, cytoplasm, nucleus, extracellular region, extracellular space, cell surface, external side of the plasma membrane, and excitatory synapse are in CC and receptor binding, growth factor activity, chemokine activity and CXCR chemokine receptor binding are in MF. KEGG pathways were enriched in cytokine–cytokine receptor interaction, pathways in cancer, viral protein interaction with cytokine and cytokine receptor, NF-kappa B signaling pathway, chemokine signaling pathway, lipid and atherosclerosis, rheumatoid arthritis, AGE-RAGE signaling pathway in diabetic complications, amoebiasis, and TNF signaling pathway. KEGG enrichment was associated with inflammation-related pathways. An inflammatory cascade following LVAD implantation is essential. Functional enrichment analysis demonstrated that cytokine-related receptor interaction was a consequential pathway detected as a potential candidate for recent polygenic human adaptation (19). Grosman-Rimon et al. found that inflammatory markers increased over time with long-term LVAD support (20). The results of GO and KEGG enrichment analyses in our study showed that some genes responsible for the production of inflammatory proteins were overexpressed after LVAD implantation.

The top 10 hub genes were identified using Cytohubba in this study. These are *CCL2*, *CXCL1*, *CXCL12*, *CTGF*, *CX3CR1*, *POSTN*, *FKBP5*, *SELE*, *AIF1*, and *BMP2*. We found that the expression levels of *CCL2*, *CXCL1*, *CXCL12*, and *CX3CR1* were obviously downregulated and those of *CTGF*, *POSTN*, *FKBP5*, *SELE*, *AIF1*, and *BMP2* were upregulated after LVAD implantation. The expression levels of *CCL2*, *CXCL12*, and *FKBP5* were remarkably different between pre- and post-LVAD. The three main hub genes demonstrated powerful diagnostic abilities with an AUC >0.90. *CCL2*, *CXCL12*, and *FKBP5* could be considered biomarkers for predicting and evaluating the effects of LVAD transplantation.

Our study shows that *CCL2* was regarded as one of the marvelous hub genes. The expression level of *CCL2* significantly decreased after LVAD implantation in our study. *CCL2* is an inflammatory chemokine that plays an important role in the inflammatory response cascade (21). It could induce inflammation-associated cell aggregation and stimulate the activity of monocytes and basophils after LVAD implantation (22). Grosman-Rimon et al. found that markers of inflammation remained higher than before LVAD implantation at 3, 6, and 9 months after LVAD implantation (20). *CCL2* may be involved in cardiac remolding and protects cardiomyocytes against cell death mediated by hypoxia. *CCL2* showed a powerful diagnostic ability with an AUC > 0.90. The expression level of *CCL2* seems to predict the severity of heart failure. *CCL2* could be considered an ideal biomarker for LVAD support. Li et al. reported that *CCL2* is a crucial hub gene in LVAD pathophysiology (23).

*CXCL12* is an encoded protein and chemokine ligand. It binds to a G-protein-coupled receptor and CXC receptor 4 and plays a variety of important roles in many diverse cellular functions, including immune surveillance, inflammation response, tissue homeostasis, and tumor growth and metastasis (24–27). In our study, we showed that *CXCL12* might be involved in BP, including chemokine-mediated signaling pathway, positive regulation of monocyte chemotaxis, cell adhesion, response to hypoxia, response to peptide hormone, G-protein-coupled receptor signaling pathway, positive regulation of T cell migration, positive regulation of calcium ion import, blood circulation, and positive regulation of cell migration. The MF of *CXCL12* includes growth factor activity, chemokine activity, receptor binding, and CXCR chemokine receptor binding. The AUC of *CXCL12* was also >0.90 in our study, suggesting that it has a powerful diagnostic capability. At least three independent transcriptional studies of LVADs have detected *CCL2* and *CXCL12*, indicating that mechanical unloading may influence gene expression regardless of the changes in cardiac function (28).

Multiple tissues express *FKBP5* with different distributions, which plays an important role in cellular processes. *FKBP5* exhibits peptide-prolyl isomerase activity and regulates protein folding. It influences the steroid receptor signal and NF-κB pathway. Stress may also result in the chemotaxis of proinflammatory cells and peripheral inflammation, possibly leading to a higher proinflammatory status and increased cardiovascular risk (29).

*FKBP5* regulates glucocorticoid receptors. High levels of FKBP5 reduce effective glucocorticoid receptors and accelerate glucocorticoid resistance. Glucocorticoid resistance weakens the compensatory ability of the heart under stressful conditions. The expression of FKBP5 is fundamentally associated with stress-related diseases. When exposed to high pressure, patients exhibit high FKBP5 expression and are prone to inflammation and acute cardiovascular events (29). The *FKBP5* gene product is a potential biomarker and therapeutic agent for heart failure (30) and may be used to diagnose heart failure with an LVAD. FKBP5 displayed a high degree of diagnostic ability with an AUC of 0.9977.

Bone morphogenetic protein-2 (BMP2) is a low-molecular-weight glycoprotein classified as a morphogen. It belongs to the transforming growth factor-β (TGF-β) superfamily. *BMP2* plays an important role in vascular biology (31, 32), and increased *BMP-2* levels can promote inflammation and atherosclerosis through oxidative stress and endothelial dysfunction. *BMP2* is involved in inflammation and fibrogenesis during heart remolding. *BMP2* also demonstrated a high diagnostic ability in our study, with an AUC of 0.8701.

LVAD support time was longer for high-expression *CCL2* and *CXCL12* groups than that for low-expression groups, while it was shorter for the high-expression *FKBP5* and *BMP2* groups in our study. However, there was no statistically significant difference in the duration between the groups with high and low expression levels (*P *> 0.05). LVEDD, LVEF, and CI showed no difference in hub gene expression levels.

The individuals included in our study were patients with DCM who underwent heart transplantation after LVAD implantation. LVAD unloading reduced the levels of inflammatory factor transcripts in patients with DCM. *CCL2* and *CXCL12* expression was low, whereas *FKBP5* and *BMP2* expression was high. This suggests reverse remodeling of the myocardium caused by mechanical unloading or deterioration of the patient's condition. These genes could be considered biomarkers for predicting and evaluating the effects of LVAD implantation in patients with DCM.

Despite discovering some potential genes associated with LVAD support at the end stage of CHF from DCM, the bioinformatics analysis of the study has some limitations. The number of patients investigated was small. We obtained only 28 paired myocardium microarrays from patients with DCM who underwent LVAD implantation and heart transplantation. It is not easy to obtain microarray datasets from the myocardium of patients with DCM who underwent LVAD implantation and heart transplantation from GEO datasets. This is an inherent limitation of bioinformatics analysis. The results from a bioinformatics analysis should be further validated using a larger sample cohort. In addition, the GEO dataset did not provide sufficient clinical information for this special population. Although the bioinformatic analysis has the aforementioned shortcomings, it can still provide a better understanding of LVAD pathophysiology from a gene expression perspective.

*CCL2, CXCL12, FKBP5*, and *BMP2* could be potential gene biomarkers for patients with DCM after LVAD support. The expression level of hub genes was not statistically significant in LVEDD, LVEF, CI, and the support time of LVAD. Nevertheless, it may still be helpful to therapeutically manage DCM patients with LVADs.

## Data Availability

The datasets presented in this study can be found in online repositories. The names of the repository/repositories and accession number(s) can be found in the article/Supplementary Material.

## References

[1] Dec GW Fuster V. Idiopathic dilated cardiomyopathy. N Engl J Med. (1994) 331:1564–75. 10.1056/nejm199412083312307 7969328

[2] Paluszkiewicz L Kukulski T Zembala M Gummert J Morshuis M. The role of long-term mechanical circulatory support in the treatment of end-stage heart failure. Kardiol Pol. (2019) 77:331–40. 10.5603/KP.a2019.0027 30915780

[3] Kirklin JK Naftel DC Pagani FD Kormos RL Stevenson LW Blume ED Seventh INTERMACS annual report: 15,000 patients and counting. J Heart Lung Transplant. (2015) 34:1495–504. 10.1016/j.healun.2015.10.003 26520247

[4] Bellavia D Iacovoni A Scardulla C Moja L Pilato M Kushwaha SS Prediction of right ventricular failure after ventricular assist device implant: systematic review and meta-analysis of observational studies. Eur J Heart Fail. (2017) 19:926–46. 10.1002/ejhf.733 28371221

[5] Mehra MR Naka Y Uriel N Goldstein DJ Cleveland JC Colombo PC A fully magnetically levitated circulatory pump for advanced heart failure. N Engl J Med. (2017) 376:440–50. 10.1056/NEJMoa1610426 27959709

[6] Chen Y Park S Li Y Missov E Hou M Han X Alterations of gene expression in failing myocardium following left ventricular assist device support. Physiol Genomics. (2003) 14:251–60. 10.1152/physiolgenomics.00022.2003 12824457

[7] Schwientek P Ellinghaus P Steppan S D'Urso D Seewald M Kassner A Global gene expression analysis in nonfailing and failing myocardium pre- and postpulsatile and nonpulsatile ventricular assist device support. Physiol Genomics. (2010) 42:397–405. 10.1152/physiolgenomics.00030.2010 20460602

[8] Chen C Chen H Zhang Y Thomas HR Frank MH He Y TBtools: an integrative toolkit developed for interactive analyses of big biological data. Mol Plant. (2020) 13:1194–202. 10.1016/j.molp.2020.06.009 32585190

[9] Shen W Song Z Zhong X Huang M Shen D Gao P Sangerbox: a comprehensive, interaction-friendly clinical bioinformatics analysis platform. iMeta. (2022) 1:e36. 10.1002/imt2.36 PMC10989974 38868713

[10] Huang DW Sherman BT Lempicki RA. Bioinformatics enrichment tools: paths toward the comprehensive functional analysis of large gene lists. Nucleic Acids Res. (2009) 37:1–13. 10.1093/nar/gkn923 19033363 PMC2615629

[11] Huang DW Sherman BT Lempicki RA. Systematic and integrative analysis of large gene lists using DAVID bioinformatics resources. Nat Protoc. (2009) 4:44–57. 10.1038/nprot.2008.211 19131956

[12] von Mering C Jensen LJ Snel B Hooper SD Krupp M Foglierini M STRING: known and predicted protein–protein associations, integrated and transferred across organisms. Nucleic Acids Res. (2005) 33:D433–7. 10.1093/nar/gki005 15608232 PMC539959

[13] von Mering C Huynen M Jaeggi D Schmidt S Bork P Snel B. STRING: a database of predicted functional associations between proteins. Nucleic Acids Res. (2003) 31:258–61. 10.1093/nar/gkg034 12519996 PMC165481

[14] Snel B Lehmann G Bork P Huynen MA. STRING: a web-server to retrieve and display the repeatedly occurring neighbourhood of a gene. Nucleic Acids Res. (2000) 28:3442–4. 10.1093/nar/28.18.3442 10982861 PMC110752

[15] Bank AJ Mir SH Nguyen DQ Bolman RM Shumway SJ Miller LW Effects of left ventricular assist devices on outcomes in patients undergoing heart transplantation. Ann Thorac Surg. (2000) 69:1369–74. 10.1016/s0003-4975(00)01083-3 10881807

[16] Klotz S Jan Danser AH Burkhoff D. Impact of left ventricular assist device (LVAD) support on the cardiac reverse remodeling process. Prog Biophys Mol Biol. (2008) 97:479–96. 10.1016/j.pbiomolbio.2008.02.002 18394685

[17] Soppa GK Barton PJ Terracciano CM Yacoub MH. Left ventricular assist device-induced molecular changes in the failing myocardium. Curr Opin Cardiol. (2008) 23:206–18. 10.1097/HCO.0b013e3282fc7010 18382208

[18] Blaxall BC Tschannen-Moran BM Milano CA Koch WJ. Differential gene expression and genomic patient stratification following left ventricular assist device support. J Am Coll Cardiol. (2003) 41:1096–106. 10.1016/s0735-1097(03)00043-3 12679207

[19] Daub JT Hofer T Cutivet E Dupanloup I Quintana-Murci L Robinson-Rechavi M Evidence for polygenic adaptation to pathogens in the human genome. Mol Biol Evol. (2013) 30:1544–58. 10.1093/molbev/mst080 23625889

[20] Grosman-Rimon L Jacobs I Tumiati LC McDonald MA Bar-Ziv SP Fuks A Longitudinal assessment of inflammation in recipients of continuous-flow left ventricular assist devices. Can J Cardiol. (2015) 31:348–56. 10.1016/j.cjca.2014.12.006 25746024

[21] Dewald O Zymek P Winkelmann K Koerting A Ren GF Abou-Khamis T CCL2/monocyte chemoattractant protein-1 regulates inflammatory responses critical to healing myocardial infarcts. Circ Res. (2005) 96:881–9. 10.1161/01.RES.0000163017.13772.3a 15774854

[22] Gonzalez-Quesada C Frangogiannis NG. Monocyte chemoattractant protein-1/CCL2 as a biomarker in acute coronary syndromes. Curr Atheroscler Rep. (2009) 11:131–8. 10.1007/s11883-009-0021-y 19228487 PMC2787514

[23] Li G Chen Z Zhang Y Wu Z Zheng J. Effects of left ventricular assist device on heart failure patients: a bioinformatics analysis. Artif Organs. (2020) 44:577–83. 10.1111/aor.13627 31875973

[24] McIntosh SZ Quinn KE Ashley RL. CXCL12 may drive inflammatory potential in the ovine corpus luteum during implantation. Reprod Sci. (2022) 29:122–32. 10.1007/s43032-021-00791-0 34755321 PMC8677687

[25] Murad HAS Rafeeq MM Alqurashi TMA. Role and implications of the CXCL12/CXCR4/CXCR7 axis in atherosclerosis: still a debate. Ann Med. (2021) 53:1598–612. 10.1080/07853890.2021.1974084 34494495 PMC8439212

[26] Miao YD Wang JT Tang XL Mi DH. Microarray analysis to explore the effect of CXCL12 isoforms in a pancreatic pre-tumor cell model. World J Gastroenterol. (2021) 27:8194–8. 10.3748/wjg.v27.i47.8194 35068863 PMC8704271

[27] Baci D Bruno A Cascini C Gallazzi M Mortara L Sessa F Acetyl-L-carnitine downregulates invasion (CXCR4/CXCL12, MMP-9) and angiogenesis (VEGF, CXCL8) pathways in prostate cancer cells: rationale for prevention and interception strategies. J Exp Clin Cancer Res. (2019) 38:464. 10.1186/s13046-019-1461-z 31718684 PMC6852951

[28] Ton V-K Vunjak-Novakovic G Topkara VK. Transcriptional patterns of reverse remodeling with left ventricular assist devices: a consistent signature. Expert Rev Med Devices. (2016) 13:1029–34. 10.1080/17434440.2016.1243053 27685648

[29] Zannas AS Jia M Hafner K Baumert J Wiechmann T Pape JC Epigenetic upregulation of FKBP5 by aging and stress contributes to NF-κB-driven inflammation and cardiovascular risk. Proc Natl Acad Sci U S A. (2019) 116:11370–9. 10.1073/pnas.1816847116 31113877 PMC6561294

[30] Kolur V Vastrad B Vastrad C Kotturshetti S Tengli A. Identification of candidate biomarkers and therapeutic agents for heart failure by bioinformatics analysis. BMC Cardiovasc Disord. (2021) 21:329. 10.1186/s12872-021-02146-8 34218797 PMC8256614

[31] Rutkovskiy A Sagave J Czibik G Baysa A Enayati KZ Hillestad V Connective tissue growth factor and bone morphogenetic protein 2 are induced following myocardial ischemia in mice and humans. Scand J Clin Lab Investig. (2017) 77:321–31. 10.1080/00365513.2017.1318447 28460577

[32] Kercheva M Gusakova AM Ryabova TR Suslova TE Kzhyshkowska J Ryabov VV. Serum levels of bone morphogenetic proteins 2 and 4 in patients with acute myocardial infarction. Cells. (2020) 9:2179. 10.3390/cells9102179 32992577 PMC7601292

